# Maize/soybean intercropping facilitated phosphorus solubilization via shifted and synergistic arbuscular mycorrhizal fungal and bacterial communities in red soil

**DOI:** 10.3389/fpls.2025.1638043

**Published:** 2025-11-26

**Authors:** Ling Qian, Li Tang, Jingxiu Xiao, Lili Mao, Ping Lv, Faming Zhang, Yi Zheng

**Affiliations:** 1 College of Plant Protection, Yunnan Agricultural University, Kunming, Yunnan, China; 2 College of Resource and Environment, Yunnan Agricultural University, Kunming, Yunnan, China; 3 Institute of Resources and Environment, Kunming Academy of Agricultural Sciences, Kunming, Yunnan, China; 4 College of Agriculture and Horticultural Technology, Yunnan Vocational College of Agriculture, Kunming, Yunnan, China; 5 Environmental Testing Laboratory, Kunming Customs Technology Center, Kunming, Yunnan, China; 6 Department of President Office, Yunnan Open University, Kunming, Yunnan, China

**Keywords:** maize/soybean intercropping, arbuscular mycorrhizal fungi, red soil, phosphorus solubilization, PSB

## Abstract

**Introduction:**

Arbuscular mycorrhizal fungi (AMF) are key regulators of phosphorus (P) cycling in agricultural systems. However, under intercropping conditions, the mechanisms through which AMF hyphae and spores recruit specific bacterial taxa and synergistically solubilize insoluble P in red soils remain poorly understood.

**Methods:**

Through a greenhouse pot experiment, we investigated how the symbiotic relationship between AMF and crops varies across a gradient of P fertilizer levels (P0 to P250). We aimed to identify the P level that optimizes this symbiosis and to elucidate, via high-throughput sequencing and network analysis, the regulatory mechanism by which interactions between AMF and phosphate-solubilizing bacteria (PSB) drive P solubilization.

**Results:**

Mycorrhizal colonization rate, hyphal length density (HLD), and spore density (SD) exhibited a hump-shaped response to increasing P fertilizer inputs, peaking at P150. IMS enhanced these parameters and also enriched the AMF taxon *Glomus_f_Glomeraceae* and eight key bacterial genera (e.g., *Sphingomonas*, *Unclassified_f_Micrococcaceae*, and *Streptomyces*). The relative abundance of *Glomus_f_Glomeraceae* was highest at P150, corresponding to the strongest AMF-crop symbiosis. Network analysis revealed a higher proportion of positive associations between AMF and bacteria in IMS than in monoculture.

**Discussion:**

Our findings demonstrate that IMS facilitates P solubilization in red soil by shifting the AMF and bacterial communities toward a more synergistic state. Furthermore, our results provide a mechanistic understanding of how optimized P management in IMS can enhance AMF and bacterial cooperation to improve P use efficiency. These insights offer novel strategies for mycorrhizal function conservation and sustainable agroecosystem management.

## 1 Introduction

Phosphorus (P) is one of the most essential mineral nutrients after nitrogen, and its availability limits crop productivity in numerous agricultural systems (Raymond et al., 2021). The concentration of plant-available P in soil is typically low due to its adsorption and precipitation into insoluble compounds. This problem is particularly acute in the acidic red soils of southern China, where low pH increases the solubility of Fe and Al oxides, which facilitates phosphate precipitation and the formation of insoluble P-containing minerals (Hinsinger, 2001). Consequently, the seasonal utilization rate of P fertilizer in these soils is often less than 10% (Lu et al., 1995). To maintain or increase crop productivity, P fertilizers with varying levels of available P are routinely applied. However, these fertilizers are derived from phosphate rock, a nonrenewable resource, with approximately 80% of the global supply used for fertilizer production (Chowdhury et al., 2014; Fischer et al., 2017). At current consumption rates, these reserves are projected to face severe depletion within decades (Cordell et al., 2009; Peñuelas et al., 2013). Given this situation, there is an urgent need to develop sustainable agricultural systems that enhance P-use efficiency and reduce dependence on phosphate fertilizers. Diversified intercropping represents one such system, harnessing the biological potential of crops. This strategy has emerged as a promising solution to the dual challenge of low P-use efficiency and global P scarcity, and is consequently attracting growing research and practical attention.

Intercropping, an ancient cropping system practiced worldwide, involves simultaneously cultivating two or more crop species on the same land. Evidence shows that this system can reduce fertilizer application per unit yield by 19%–36% compared to monoculture under equivalent management (Li et al., 2020). Specifically, studies on legume/cereal intercropping have demonstrated its ability to regulate the soil P pool, enhance P availability, and promote plant P uptake (Li et al., 2007; Wang et al., 2017; An et al., 2024). Regarding the mechanisms by which intercropping enhances P use efficiency, previous studies have primarily focused on: (1) modifications in root system architecture (Zhang et al., 2016b; An et al., 2023); (2) the secretion of protons, organic acids, and phosphatases (Li et al., 2007; Latati et al., 2014; Qu et al., 2024); and (3) the enrichment of specific microbial groups in the rhizosphere (Tang et al., 2016; Chen et al., 2020). However, while previous studies have primarily focused on the plant itself and broad microbial groups, the key mechanism—how AMF in intercropping systems recruit PSB via their hyphae and spores, thereby synergizing with them to enhance the solubilization of sparingly soluble P in red soils—remains poorly understood.

Elucidating this mechanism requires a focused examination of the rhizosphere—a pivotal zone for plant-soil interactions that plays a crucial role in nutrient cycling and sustains microbial community diversity (Kuzyakov and Blagodatskaya, 2015). Within this zone, AMF are among the most critical microorganisms governing P supply dynamics (Adomako et al., 2022). Under low-P conditions, the arbuscular mycorrhizae symbiosis can contribute up to 78% of total plant P uptake (Nagy et al., 2009). Evidence from a field study indicates that not only can the establishment of fungal hyphal networks expand the host plant's range for P acquisition by 15-fold (Mai et al., 2019), but the contribution of indigenous AMF communities to crop P nutrition can also be equivalent to the application of 30 kg P ha^-1^ of chemical P fertilizer (Wang et al., 2020). However, during their long-term co-evolution with plants, AMF have lost saprotrophic functional genes responsible for mineralizing organic P (Tisserant et al., 2013; Morin et al., 2019). To compensate for this functional deficiency and maintain symbiotic stability, AMF recruit phosphate-solubilizing bacteria (PSB) to the hyphosphere through hyphal exudates—primarily glucose, fructose, and trehalose (Bharadwaj et al., 2012; Zhang et al., 2022a; Zhang et al., 2018a). This exudation strategy exhibits striking parallels with the plant root strategy of recruiting specialized microbial communities through root metabolites (e.g., organic acids and sugars) (Zhalnina et al., 2018), as both systems enhance insoluble phosphate mobilization by regulating microbial activities. Nevertheless, under low P conditions, both AMF and root hairs shape rhizosphere microbial composition, with AMF exhibiting superior regulatory capacity; however, these regulatory effects are substantially diminished under high P conditions (Zhou et al., 2022). Critically, the root and mycorrhizal pathways are tightly interconnected, achieving functional integration through dynamic coordination (Genre et al., 2020; Chu et al., 2020; Zhou et al., 2022).

AMF serve as critical mediators of interspecific interactions (Duchene et al., 2017; Liu et al., 2021). A single AMF strain can colonize multiple host plants, while multiple strains may coexist within a single host, collectively forming an underground “common mycorrhizal network (CMN)” that interconnects diverse plant species (Babikova et al., 2013; Genre et al., 2020). Legume/cereal intercropping enhances the symbiotic relationship between crops and AMF, increasing mycorrhizal colonization rates (Zhao et al., 2020; Song et al., 2021; Zhang et al., 2024), as well as hyphal length density (HLD)and spore density (SD) in rhizosphere soil (Zhao et al., 2020; Li et al., 2023). Notably, AMF inoculation in maize/soybean intercropping (IMS) enhances the mobilization of insoluble P in red soil compared to monoculture maize (MM), thereby improving P uptake efficiency in intercropped plants (Zhong et al., 2018). This phenomenon may be associated with AMF-mediated microbial recruitment mechanisms. Beyond the recruitment of PSB through hyphal exudates, specific bacterial taxa such as *Arthrobacter* and *Streptomyces* have been found to be enriched on the surface of AMF spores (Agnolucci et al., 2015). It remains unclear whether the enhanced symbiotic relationship between crops and AMF in intercropping systems synergistically improves P activation efficiency in red soil by expanding the PSB community through dual recruitment mechanisms (hyphal exudates and spore adhesion). However, current research predominantly focuses on AMF–PSB interactions in monoculture systems, whereas the mechanisms by which the plant–AMF–bacteria continuum enhances P utilization and interacts with P fertilizer levels in intercropping systems remain elusive. Addressing this knowledge gap is critical for advancing sustainable agriculture in red soil regions. The key challenge lies in optimizing P fertilizer inputs to harness the biological potential of crops, thereby enhancing agricultural benefits, while minimizing adverse impacts on mycorrhizal functions.

We hypothesize that maize/soybean intercropping enhances the crop–AMF symbiosis, thereby increasing HLD and SD, and restructuring the AMF–bacterial interaction network in the rhizosphere microdomain. This functional integration ultimately leads to a synergistic improvement in the activation efficiency of sparingly soluble P in red soil. To test this hypothesis, we employed a combination of a pot experiment and high-throughput sequencing to systematically investigate how AMF and bacterial communities respond to and interact under varying P fertilizer levels and cropping systems. The primary objectives were to: (1) quantify the regulatory effects of P fertilizer levels and cropping systems on mycorrhizal colonization rates, HLD, and SD; (2) decipher the AMF–bacterial interaction network to elucidate the key microbial mechanisms that synergistically drive soil P activation; and (3) identify the optimal P fertilizer level threshold to optimize this process, ultimately creating optimal soil conditions for crop P utilization efficiency.

## 2 Materials and methods

### 2.1 Experiment site and materials

The study was conducted from July to September 2023 in a greenhouse under natural daylight conditions at the experimental field of Yunnan Agricultural University, Kunming, China (102°45′4″ E, 25°8′1″ N). The pot experiment utilized red soil collected from Xiaoshao Village in the Guandu District of Kunming. This soil, classified as an Ultisol according to USDA soil taxonomy, is representative of the typical red soils of southern and southwestern China. The initial characteristics of this soil were as follows: pH 5.56, organic matter 10.15 g·kg^−1^, available nitrogen 56.15 mg·kg^−1^, available P 5.50 mg·kg^−1^, and available potassium 79.02 mg·kg^−1^ (detailed physicochemical properties are provided in **Supplementary Table S1**). During the experimental period, the ambient temperature ranged from 22 to 35°C, with a photoperiod of 10 to 12 h/day. The test plants, maize (*Zea mays* L.) cultivar 'Yunrui 88' and soybean (*Glycine max* (L.) Merr.) cultivar 'Yunhuang 13', were obtained from the Yunnan Academy of Agricultural Sciences.

### 2.2 Experimental design

A two-factor experiment was employed, involving P fertilizer levels and cropping systems. The P levels were determined based on the results of a preliminary experiment, in which the P150 treatment exhibited the highest mycorrhizal colonization rates, HLD, and SD in rhizosphere soil (data not shown). The tested P fertilizer levels included a no-P control (P0) and five application rates: 50, 100, 150, 200, and 250 mg P_2_O_5_ kg^−1^ dry soil (denoted by P50, P100, P150, P200, and P250, respectively). The cropping systems included MM, monoculture soybean (MS), and IMS. With all six P levels applied to each of the three cropping systems, the experiment consisted of a total of 18 treatments (**Supplementary Table S2**), each replicated four times.

Urea (CO(NH_2_)_2_, 46% N), single superphosphate (16% P_2_O_5_), and potassium sulfate (50% K_2_O) were applied as base fertilizers for N, P, and K, respectively, before sowing. The application rates were 200 mg kg^−1^ dry soil for both N and K_2_O. Prior to planting, the soil was homogenized, and stones and plant residues were removed. Maize and soybean seeds of uniform size and plumpness were selected, surface-sterilized with 1% hydrogen peroxide for 3 min, and thoroughly rinsed with sterile water. The seeds were sown in a plastic pot (height: 230mm × diameter: 250mm), each containing 10kg of soil, on 20 July 2022. After emergence, the seedlings were thinned to retain two maize seedlings per pot for MM, two soybean seedlings for MS, or one maize and one soybean seedling for IMS. To minimize experimental error, all pots were managed uniformly under consistent conditions and randomly repositioned at regular intervals during the growth period. Irrigation and weed management were adjusted based on plant growth observations. Pesticides were not applied throughout the experiment to avoid disturbing soil microbial activity.

### 2.3 Sampling and sample processing

Rhizosphere soil and plant samples were collected on 7 September 2023, which was 48 days after sowing and corresponded to the maize V12 stage. Simultaneously, the roots were shaken slightly to remove the bulk soil, and then the rhizosphere soil adhering to the whole roots was brushed off and homogenized. The collected soil was sieved (2mm) and divided into two subsamples. One subsample of soil was stored at − 80°C for subsequent microbial DNA extraction, while the other was air-dried for the determination of HLD and soil physicochemical properties. The roots were then carefully rinsed with water to remove residual soil and stored at 4°C for the assessment of AMF colonization in the roots. Soil available P (AP, measured as Olsen-P), total P (TP), and available nitrogen (AN) were determined as follows: AP by the Olsen method, TP by the sodium hydroxide (NaOH) fusion method, and AN by the diffusion adsorption method (Bao, 2000). AMF colonization in the roots was quantified using the trypan blue staining method (Giovannetti and Mosse, 1980). The HLD was measured in the soil using the membrane filter technique (Jakobsen et al., 1992). AMF spores were extracted from 10.0g of air-dried soil using the wet sieving and decanting method (Ianson and Allen, 1986) and counted under a stereomicroscope. SD was expressed as the number of spores per gram of air-dried soil.

### 2.4 Soil DNA extraction and sequencing

Following the manufacturer’s instructions, soil microbial DNA was extracted from 0.5g of frozen rhizosphere soil using the FastDNA Spin Kit for Soil (MP Biomedicals, Santa Ana, California, United States of America). The quality and concentration of the DNA were assessed by 1.0% agarose gel electrophoresis and a NanoDrop 2000 spectrophotometer (Thermo Scientific, Waltham, Massachusetts, United States of America), respectively. The purified DNA was stored at − 80°C for subsequent analysis.

The V4–V5 hypervariable region of the 18S rRNA gene and the V3–V4 hypervariable region of the 16S rRNA gene were used to amplify the sequences of AMF and bacteria in the sample DNA, respectively. A nested PCR approach was employed for the AMF 18S rRNA gene region. The first round of amplification used the universal primers AML1F (5′-ATCAACTTTCGATGGTAGGATAGA-3′) and AML2R (5′-GAACCCAAACACTTTGGTTTCC-3′). This was followed by a second round of amplification using the AMF-specific primers AMV4-5NF (5′-AAGCTCGTAGTTGAATTTCG-3′) and AMDGR (5′-CCCAACTATCCCTATT AATCAT-3′). Amplicon sequencing was performed on the Illumina MiSeq PE300 platform by Majorbio Bioinformatics Technology Co. Ltd. (Shanghai, China).

### 2.5 Processing of sequencing data

The raw sequencing data were quality-filtered using FASTP (v0.19.6) (Chen et al., 2018), and the paired-end clean reads were merged using FLASH (v1.2.11) (Magoč and Salzberg, 2011). The resulting optimized sequences were clustered into operational taxonomic units (OTUs) at a 97% sequence similarity threshold using UPARSE (v11.0) (Stackebrandt and Goebel, 1994; Edgar, 2013). Taxonomic annotation was assigned to each OTU representative sequence with a confidence threshold of 0.7, based on the MaarjAM database (v.81) for AMF (Öpik et al., 2010) and the Silva (v.138) database for bacteria (Wang et al., 2007), respectively.

### 2.6 Data analysis

The P activation coefficient (PAC), which reflects the transformation between soil total P and available P (Yang et al., 2019), was calculated as follows:


PAC(%)=[AP/(TP×1000)]×100

Where AP represents the soil available P content (mg kg^−1^) and TP denotes total P content (mg kg^−1^).

The statistical analyses were conducted by IBM SPSS Statistics 24.0. Prior to analysis of variance (ANOVA), the assumptions of normality and homogeneity of variances were assessed using the Shapiro–Wilk test and Levene’s test, respectively. For data satisfying both assumptions, A two-way ANOVA followed by Duncan’s multiple comparison test was performed to evaluate the effects of P fertilizer level and cropping system, as well as their interaction (*p <*0.05), on mycorrhizal colonization parameters, soil properties, and the diversity of both AMF and bacterial communities. Additionally, one-way ANOVA coupled with Duncan’s test was conducted to compare treatment means at a significance level of *p <*0.05. The sequencing data analysis was conducted using the Majorbio Cloud Platform. Alpha diversity indices (including the Shannon and ACE indices) were calculated based on an OTU table using the Mothur software (v.1.30.2). Nonmetric multidimensional scaling (NMDS) was performed based on the Bray–Curtis distance matrix using the vegan package (version 2.4.3) in R software. All bar graphs were plotted, and all regression analyses were performed using GraphPad Prism software (v.10.0.0).

To elucidate the interactions between AMF and bacterial communities in rhizosphere soil, an interdomain ecological network was constructed using Networkx (v1.11). Only OTUs with a prevalence of ≥ 10 (i.e., present in 10 or more samples) were retained. Spearman correlation coefficients were calculated for all OTU pairs and co-occurrence relationships between OTUs were identified based on a threshold of |*r*|≥ 0.60 and *p <*0.05 (Barberán et al., 2012). Key network properties, including the number of edges, number of nodes, average degree, average path length, network density, network diameter, and clustering coefficient, were calculated. The significant co-occurrences network was mapped and visualized using Gephi (v0.10.1) (Bastian et al., 2009). Furthermore, Pearson correlation analysis was conducted to assess the relationships between the relative abundances of the top 12 bacterial genera and AMF parameters (Shannon index, ACE index, mycorrhizal colonization rate, HLD, and SD), as well as soil chemical properties (TP, AP, PAC, and AN/AP). The resulting correlation heatmap was visualized using the ChiPlot online platform (https://www.chiplot.online/).

## 3 Results

### 3.1 Mycorrhizal colonization rate, HLD, SD, and P availability of the rhizosphere soil

**Figure 1:**
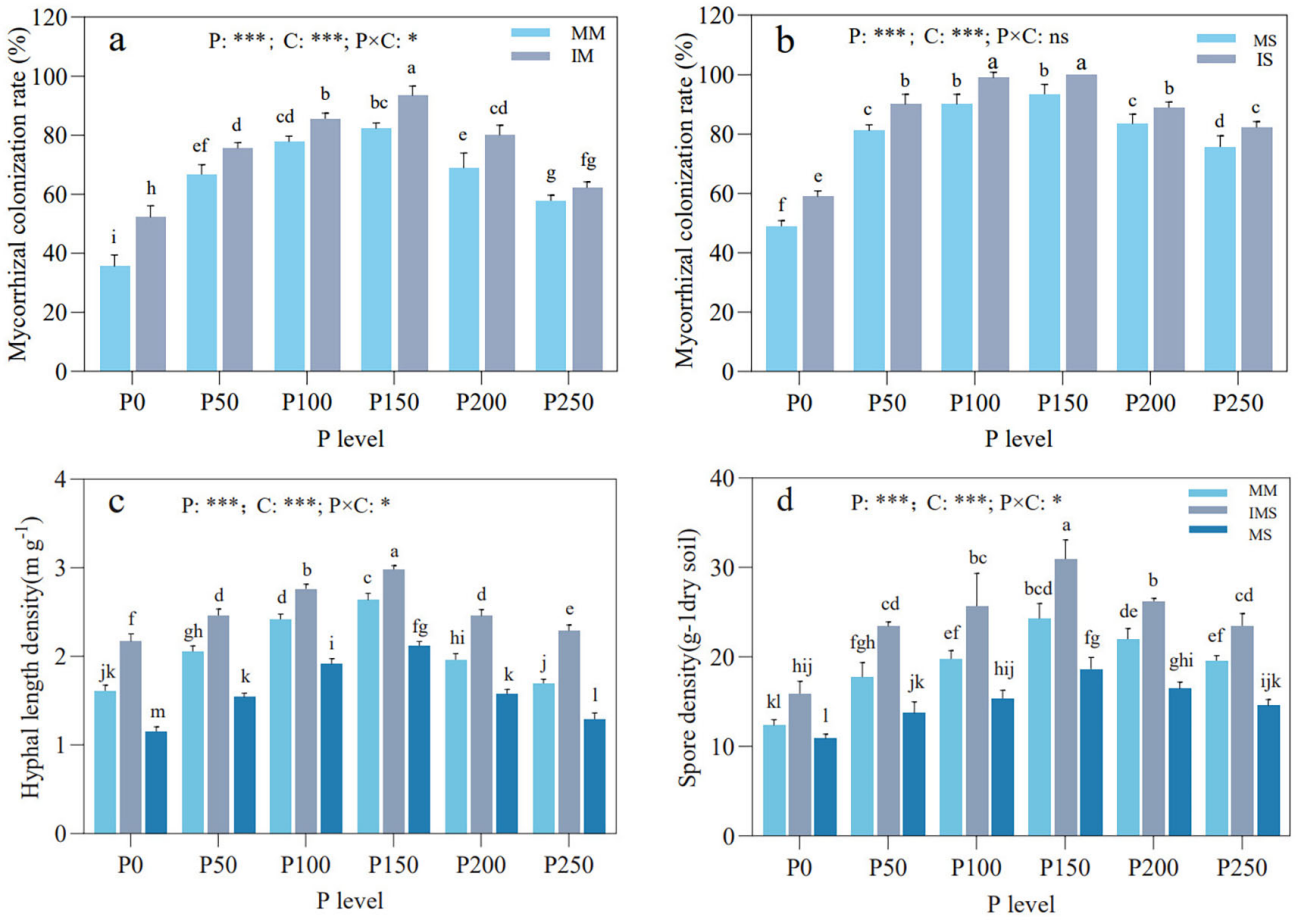
Mycorrhizal colonization rates in maize **(a)** and soybean **(b)** roots, and HLD **(c)** and SD **(d)** in rhizosphere soil under different P fertilizer levels and cropping systems. Different lowercase letters indicate significant differences among different treatments (Duncan’s multiple range test, *p* < 0.05). *p < 0.05; ** *p* < 0.01; *** *p* < 0.001. P, P fertilizer levels; C, cropping system.

Mycorrhizal colonization rates in maize and soybean, along with HLD and SD in the rhizosphere soil, exhibited a hump-shaped trend with increasing P fertilizer inputs, peaking at the P150 level (**Figure 1**). In IMS, mycorrhizal colonization rates for both maize and soybean were significantly higher than those in their respective monoculture, except for maize at the P250 level (**Figures 1a, b**). At any given P level, HLD and SD in rhizosphere soil consistently followed the order of IMS > MM > MS among the cropping systems (**Figures 1c, d**). Both P fertilizer level and cropping system exerted a significant impact on mycorrhizal colonization rates in maize and soybean, along with HLD and SD in the rhizosphere soil. Moreover, their interaction also significantly affected mycorrhizal colonization in maize, HLD, and SD.

**Figure 2:**
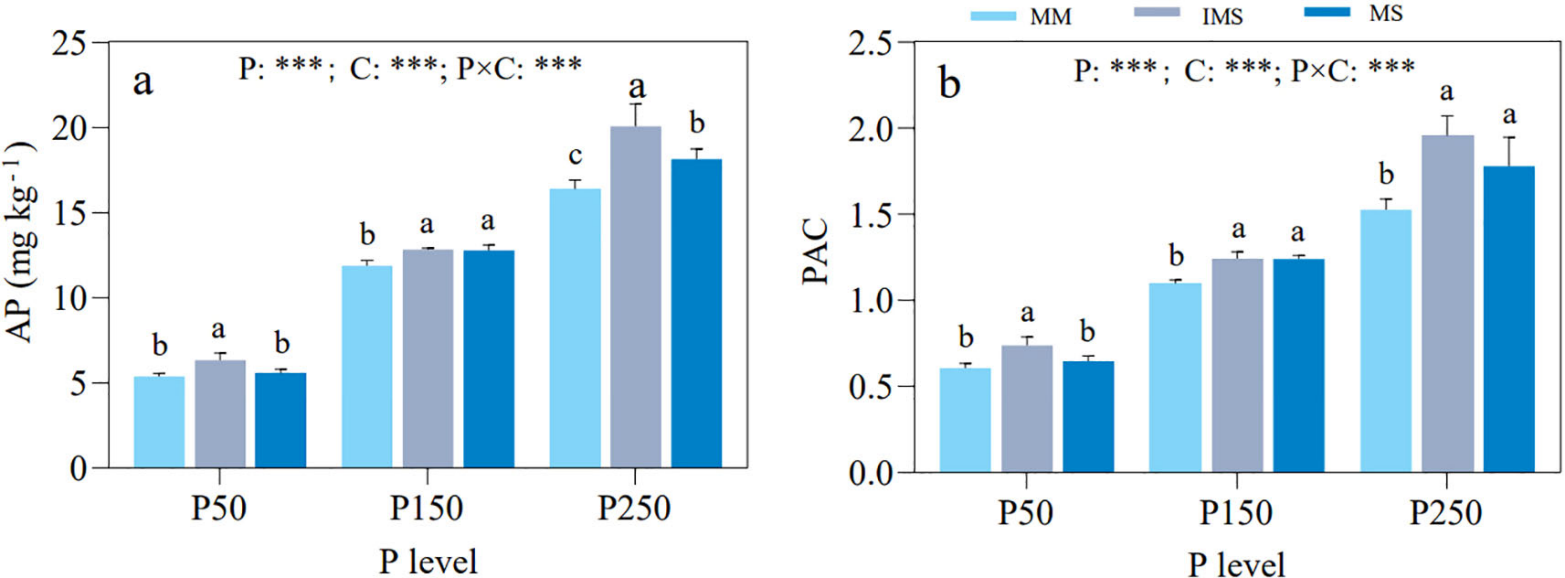
AP **(a)** and PAC **(b)** in rhizosphere soil under different P fertilizer levels and cropping systems. Note: different lowercase letters indicate significant differences among cropping systems at the same P fertilizer level (Duncan’s multiple range test, *p* < 0.05). ^*^p < 0.05; ^**^p < 0.01; ^***^p < 0.001. P, P fertilizer levels C, cropping system.

Based on the response patterns of mycorrhizal colonization rates in maize and soybean, along with HLD and SD in rhizosphere soil, three P fertilizer levels (P50, P150, and P250) were selected for determining soil AP and TP, and for calculating the PAC. The results showed that AP and PAC were significantly influenced by P input level, cropping system, and their interaction. At the same P level, IMS exhibited significantly higher AP and PAC than MM. Although IMS also showed higher values than MS, the differences were not always statistically significant (**Figure 2**).

### 3.2 Diversity and composition of AMF and bacterial communities

**Figure 3:**
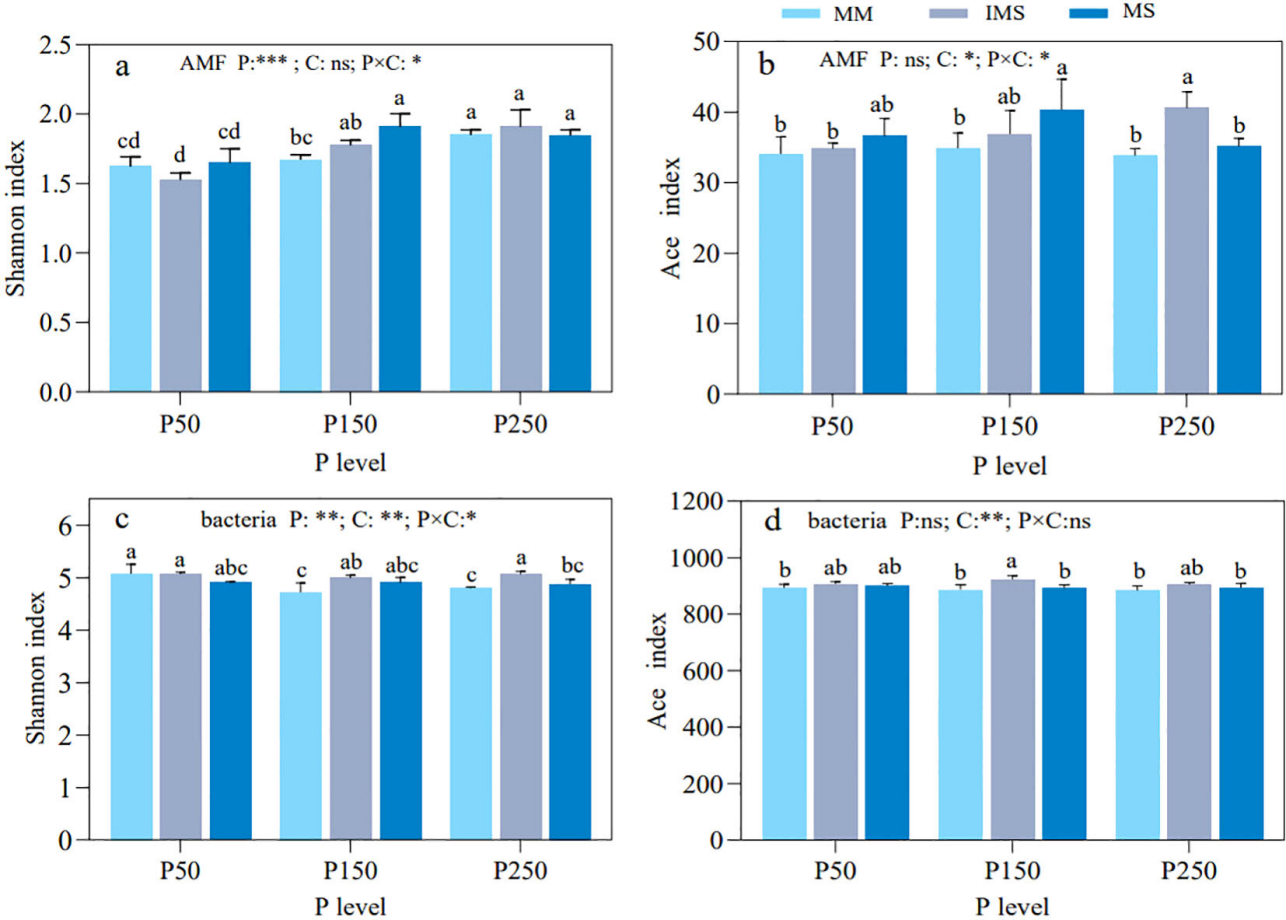
Alpha diversity of AMF **(a, b)** and bacterial **(c, d)** communities in rhizosphere soil under different P fertilizer levels and cropping systems. Shannon and Ace indices were used to characterize the alpha diversity. Note: different lowercase letters indicate significant differences (Duncan’s multiple range test, *p* < 0.05). ^*^p < 0.05; ^**^p < 0.01; ^***^p < 0.001; ns, not significant at *α* = 0.05. P, P fertilizer levels C, cropping systems.

P fertilizer levels had a significant effect on the diversity of AMF in rhizosphere soil (**Figures 3a, b**). The Shannon indices of AMF in both MM and IMS treatments showed an increasing trend with increasing P fertilizer inputs. In contrast, the Shannon index in MS increased first and then decreased from P50 to P250, peaking at P150. The Shannon index of AMF in IMS was consistently higher than in MM at the same P fertilizer level, except at P50, although the differences were not significant (**Figure 3a**). Meanwhile, the richness index of AMF (Ace) exhibited an increasing but non-significant trend with higher P fertilizer inputs, and it was markedly shaped by cropping system and its interaction with P fertilizer level (**Figure 3b**).

Compared with AMF, the Shannon index of rhizosphere bacteria responded significantly to P fertilizer level, cropping system, and their interaction (**Figures 3c, d**). The Shannon index of bacteria in MM decreased significantly with increasing P fertilizer inputs, while there was no significant change in IMS and MS. Similarly, at the same P fertilizer level, the Shannon index of bacteria in IMS (except at P50) was significantly higher than that in MM by 5.85% and 5.52%, respectively for P150 and P250 (**Figure 3c**). The richness index (Ace) of rhizosphere bacteria in MM and MS showed non-significant declines with increasing P fertilizer inputs, while IMS exhibited a hump-shaped trend from P50 to P250, peaking at P150. Across equivalent P fertilizer levels, the richness index in IMS was consistently higher than in both MM and MS (**Figure 3d**).

**Figure 4:**
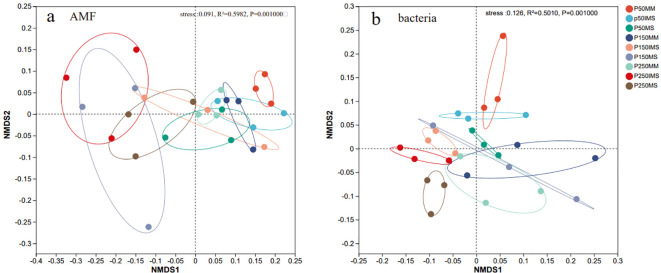
Beta diversity of AMF **(a)** and bacterial **(b)** communities in rhizosphere soil under different P fertilizer levels and cropping systems. NMDS analysis based on Bray-Curtis metrics was performed to characterize beta diversity, with differences tested via Adonis tests.

The AMF and bacterial community composition were further analyzed using NMDS based on the Bray-Curtis distance. The results indicated that the community composition of both AMF (ANOSIM: R = 0.5982, *p* ≤ 0.001; **Figure 4a**) and bacteria (ANOSIM: R = 0.5010, *p* ≤ 0.001; **Figure 4b**) was significantly influenced by P fertilizer levels and cropping systems. When P fertilizer levels were equivalent, the AMF and bacterial communities in IMS were separated from those in MM and MS. Similarly, under the same cropping system, communities were clearly separated across different P fertilizer levels (**Figure 4**). These results indicated that both P fertilizer level and cropping system collectively shaped the composition of AMF and bacterial communities.

### 3.3 Relationship between AMF community structure and symbiotic function

**Figure 5:**
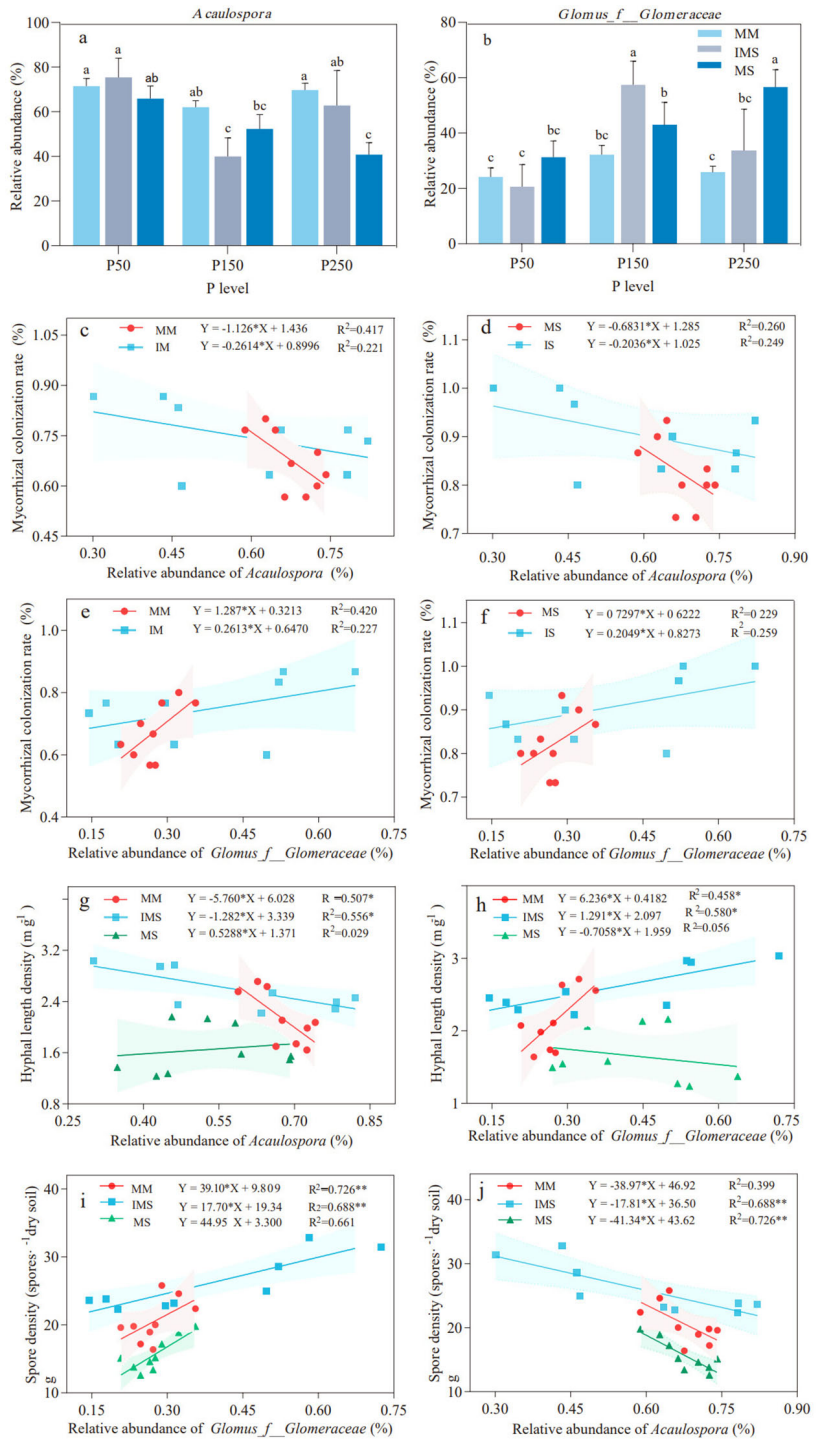
Relative abundance of dominant AMF genera and their relationships with mycorrhizal parameters under different P fertilizer levels and cropping systems. **(a, b)** Relative abundance of *Acaulospora***(a)** and *Glomus_f_Glomeraceae***(b)**. **(c–f)** Linear regression analysis between mycorrhizal colonization rate and the relative abundance of *Acaulospora* in maize **(c)** and soybean **(d)**, and of *Glomus_f_Glomeraceae* in maize **(e)** and soybean **(f)**. **(g, h)** Regression analysis of HLD versus the relative abundance of *Acaulospora***(g)** and *Glomus_f_Glomeraceae***(h)**. **(i, j)** Regression analysis of SD versus the relative abundance of *Acaulospora***(i)** and *Glomus_f_Glomeraceae***(j)**. Different lowercase letters indicate significant differences (Duncan’s multiple range test, *p* < 0.05). The shaded areas represent the 95% confidence intervals of the fitted lines. The significance of the regression equations is denoted as *p < 0.05 and **p < 0.01.

In this study, the rhizosphere AMF communities across all treatments were predominated by *Acaulospora* and *Glomus_f_Glomeraceae*, with their combined relative abundance exceeding 95% of the total community (**Supplementary Figure S1**). Compared to MM, IMS reduced the relative abundance of *Acaulospora* (except at P50) and increased that of *Glomus_f_Glomeraceae*. At the P150 level, *Acaulospora* exhibited the lowest relative abundance (35.73% and 23.63% reduction relative to MM and MS, respectively), while the relative abundance of *Glomus_f_Glomeraceae* reached its peak, exhibiting significant increases by 78.17% and 33.90% compared with MM and MS (*p <*0.05), respectively (**Figures 5a, b**).

Linear regression analysis revealed that the relationships between the abundance in dominant rhizospheric genera and mycorrhizal colonization rate, HLD, and SD varied with cropping system and P fertilizer inputs. The relative abundance of *Acaulospora* was negatively correlated with mycorrhizal colonization rates in both maize and soybean (**Figures 5c, d**), while the relative abundance of *Glomus_f_Glomeraceae* showed a positive correlation with mycorrhizal colonization rates, though neither correlation reached statistical significance (**Figures 5e, f**). The relative abundance of *Acaulospora* exhibited a significant negative correlation with HLD in MM and IMS (*p <*0.05), while showing a nonsignificant positive correlation with HLD in MS (**Figure 5g**). In contrast, the relative abundance of *Glomus_f_Glomeraceae* was positively correlated with HLD in MM and IMS, with a significant correlation in IMS (*p <*0.05). In MS, however, it showed a non-significant negative correlation with HLD (**Figure 5h**). The relative abundance of *Acaulospora* was negatively correlated with SD across all treatments, with significant correlations observed in IMS and MS treatments (*p <*0.01), whereas the relative abundance of *Glomus_f_Glomeraceae* was significantly and positively correlated with SD in all treatments (*p <*0.01) (**Figures 5i, j**).

### 3.4 Interactions between AMF and bacteria

**Figure 6:**
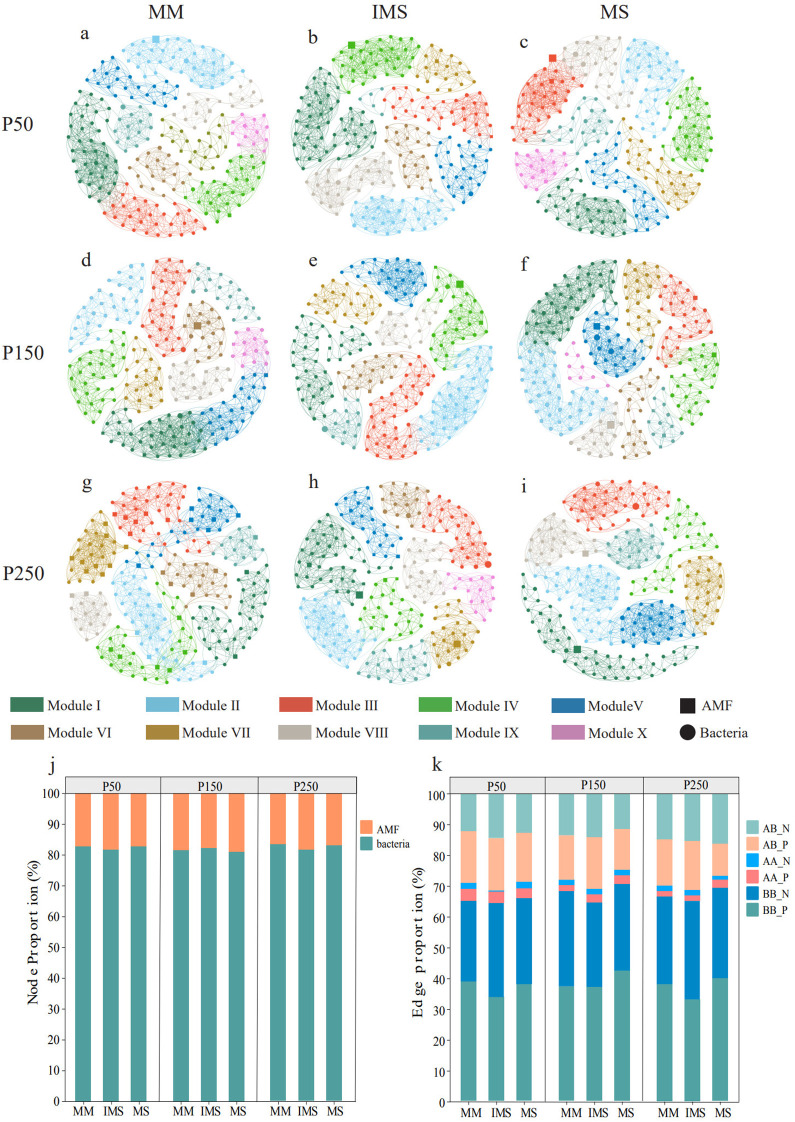
Interaction network between AMF and bacterial communities **(a–i)**. Proportions of nodes and edges in the AMF–bacteria network **(j**, **k)**. AB_N, AMF–bacteria negative associations; AB_P, AMF–bacteria positive associations; AA_N, AMF–AMF negative associations; AA_P, AMF–AMF positive associations; BB_N, bacteria–bacteria negative associations; BB_P, bacteria–bacteria positive associations.

We constructed an AMF-bacteria cross-domain ecological network to analyze the synergistic effects of P fertilizer levels and cropping systems on microbial interactions (**Figure 6**; **Supplementary Table S2**). Across all treatments, regardless of the P fertilizer level or cropping system, the co-occurrence networks between AMF and bacteria were dominated by positive interactions, indicating close ecological cooperation between these communities (**Supplementary Table S2**). Bacterial nodes exhibited dominance within the microbial interaction networks across all treatment groups (**Figure 6g**), and mutually beneficial relationships prevailed among bacterial-bacterial, AMF-AMF, and bacterial-AMF associations (**Figure 6k**). At the P50 fertilizer level, the proportion of positive bacterial-bacterial associations in IMS accounted for 33.62%, lower than those in MM (38.70%) and MS (37.84%). In contrast, the proportion of positive AMF-bacterial associations (16.93%) was slightly higher than those in MM (16.75%) and MS (16.07%) (**Figures 6a-c**), indicating that IMS enhances cross-domain cooperative partnerships between AMF and bacteria. Furthermore, this trend persisted at P150 and P250 levels, demonstrating that the regulatory effect of IMS on microbial cross-domain interactions remains stable across different P fertilizer levels (**Figures 6d-i**). As P fertilizer inputs increased, the proportion of positive AMF-bacterial associations exhibited a consistent decreasing trend in both IMS and MS networks. In comparison, this proportion declined initially and then increased in MM. Regarding bacterial-bacterial associations, their proportion displayed a hump-shaped trend in both IMS and MS, reaching a peak at the P150 level, while in MM, it declined initially and then increased (**Figure 6k**).

### 3.5 Key taxa of bacterial communities facilitated P solubilization

**Figure 7:**
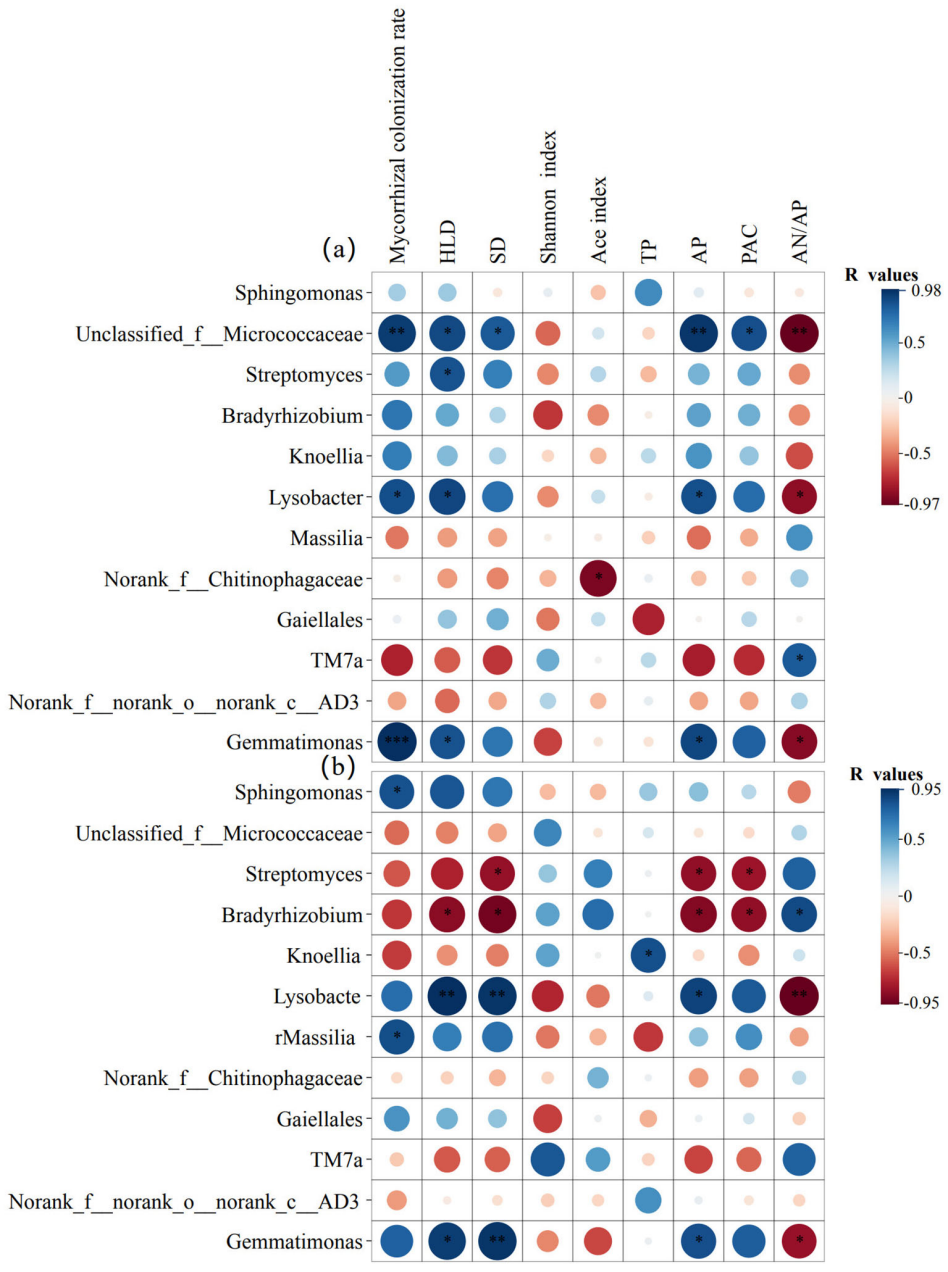
Pearson correlation heatmap. Analysis between the relative abundance of the top twelve bacterial genera and AMF parameters (diversity indices, colonization rate, HLD, SD) as well as soil chemistry (TP, AP, PAC, AN/AP) in the rhizosphere soil of maize **(a)** and soybean **(b)** under P50 conditions. The color and size of the circles represent the value of the correlation coefficient (R). ^*^p < 0.05; ^**^p < 0.01; ^***^p < 0.001.

To determine which potential bacterial taxa were involved in the activation of insoluble phosphates in the rhizosphere soil, we first analyzed the bacterial community composition (**Supplementary Figure S2**). We then performed Pearson correlation analysis between the relative abundance of the top 12 bacterial genera and parameters related to AMF and to P activation. The results showed that, compared to P50 and P150 levels, the correlations between the relative abundance of bacterial genera and characteristic parameters (including mycorrhizal colonization rate, HLD, SD, Shannon index, and Ace index) were weakened at the P250 level (**Figure 7**, **Supplementary Figures S3**, **S4**). At the P50 level, six bacterial taxa—*Unclassified_f__Micrococcaceae*, *Streptomyces*, *Bradyrhizobium*, *Knoellia*, *Lysobacter*, and *Gemmatimonas*—among the top 12 genera in maize systems (monoculture and intercropping) showed positive correlations with available P content and the P activation coefficient. Furthermore, their abundance changes were regulated by mycorrhizal colonization rate, HLD, and SD (**Figure 7a**). Similarly, four bacterial taxa—*Sphingomonas*, *Lysobacter*, *Massilia*, and *Gemmatimonas*—among the top 12 genera in soybean systems (monoculture and intercropping) were also positively correlated with available P and P activation coefficient (**Figure 7b**). Collectively, these results indicate that the above-mentioned taxa may be key players in the activation of insoluble phosphates in the rhizosphere. They further reveal that enhanced synergy between AMF and bacteria under low-P stress (at the P50 level) promotes P activation, whereas high P fertilizer conditions, as exemplified by the P250 level, inhibit this function by weakening these interkingdom interactions.

**Figure 8:**
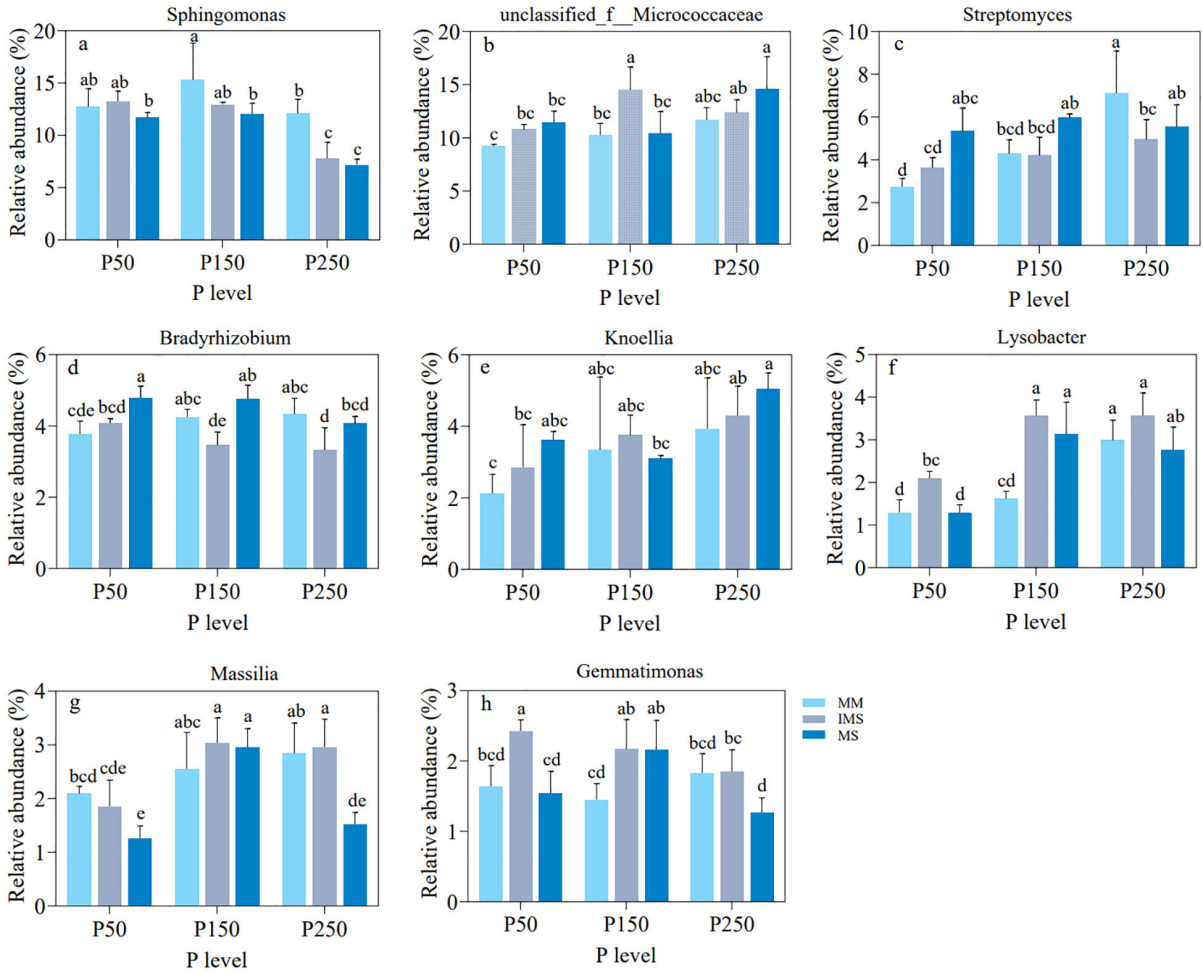
Comparison of the relative abundance of eight key bacterial genera **(a–h)** in rhizosphere soil under different P fertilizer levels (P50, P150, P250) and cropping systems (MM, IMS, MS). Different lowercase letters indicate significant differences among treatments (Duncan’s multiple range test, *p* < 0.05).

We then compared the relative abundances of the aforementioned eight bacterial genera across different treatments (**Figure 8**). At the P50 level, IMS increased the relative abundances of seven genera—*Sphingomonas*, *Unclassified_f__Micrococcaceae*, *Streptomyces*, *Bradyrhizobium*, *Knoellia*, *Lysobacter*, and *Gemmatimonas*—compared to MM, with increases ranging from 3.97% to 61.48%. At the P150 and P250 levels, five genera—*Unclassified_f_Micrococcaceae*, *Knoellia*, *Lysobacter*, *Massilia*, and *Gemmatimonas*—still maintained stable enrichment in IMS. Notably, *Massilia* in IMS consistently showed higher abundance than in both MM and MS at all P levels other than P50 (**Figure 8g**). Furthermore, in IMS, the abundances of these key taxa responded differently to increasing P fertilizer inputs: *Sphingomonas*, *Bradyrhizobium*, and *Gemmatimonas* decreased; *Unclassified_f__Micrococcaceae* and *Massilia* displayed a hump-shaped trend, peaking at the P150 level; while *Streptomyces*, *Knoellia* and *Lysobacter* increased continuously. In summary, our analysis of these key taxa revealed their consistent enrichment in IMS and their diverse responses to P fertilizer inputs. Their abundance dynamics were closely associated with both AMF dynamics and soil P availability, indicating that these taxa may collectively facilitate P activation in IMS.

## 4 Discussion

### 4.1 Effects of intercropping and P fertilizer level on mycorrhizal colonization, HLD, and SD

The symbiotic interaction between plants and AMF plays a vital role in sustaining soil ecosystem functioning and plant productivity (Van Geel et al., 2017). In this process, soil P availability represents one of the most critical environmental factors regulating mycorrhizal symbiosis (Smith and Read, 2008). Generally, extremely low soil P levels inhibit mycorrhizal colonization (Douds et al., 1998; Bolan et al., 1984). Conversely, excessive P supply suppresses symbiont formation (Liu et al., 2022; Ma et al., 2023), extraradical hyphal growth (Abbott et al., 1984), and spore development (Urcoviche et al., 2015). Consistent with these foundational principles, our results demonstrated that in a P-deficient red soil, the mycorrhizal colonization rates of maize and soybean, along with HLD and SD in the rhizosphere, exhibited a unimodal response to increasing P fertilizer inputs, peaking at the P150 level (**Figures 1**, **2**). This indicates that AMF activity is regulated by a threshold of soil P availability: low P levels restrict both plant and fungal growth, whereas high P levels (P200–P250) reduce plant dependence on AMF and its carbon allocation to the fungi (Johnson, 2010; Konvalinková et al., 2017). The optimal symbiotic relationship observed under the P150 treatment confirms that precision management of P fertilization can maximize the P acquisition function mediated by AMF (Zhang et al., 2021; Wang et al., 2020).

When compared at the same P fertilizer level, IMS significantly increased the mycorrhizal colonization rates in both crops (except for maize under P200 and P250 treatments; **Figures 1a, b**). This finding is consistent with previous studies (Zhao et al., 2020; Zhang et al., 2024). This facilitative effect may be attributed to crop diversity expanding the range of AMF host selection (Torrecillas et al., 2012; Hiiesalu et al., 2014), while alterations in root exudates provide AMF with abundant carbon sources (e.g., soluble sugars and organic acids), thereby promoting AMF colonization in both maize and soybean roots (Zhang et al., 2024). Furthermore, symbiotic signaling compounds secreted by neighboring soybean—such as flavonoids and strigolactones—stimulate AMF spore germination, hyphal growth, and branching (Sugiyama, 2019), consequently enhancing the symbiotic response of host plants (Waters et al., 2017; Campos-López et al., 2022). Notably, the competitive advantage of maize in soil nutrient acquisition reduces available P in the soybean rhizosphere. The resulting P stress enhances soybean dependence on AMF, thereby further promoting AMF colonization. The results of this study also revealed that both HLD and SD in the rhizosphere soil followed the order: IMS > MM > MS (**Figures 2a, b**). This pattern may be attributed to the heterogeneous rhizosphere environment created by crop diversity, which provides AMF with more diverse ecological niches and thereby promotes the development of their network diversity (Ahammed and Hajiboland, 2024). Additionally, as the root biomass of host plants directly influences AMF spore formation (Li et al., 2013), the significant advantages in above- and belowground biomass exhibited by intercropped maize (data not shown) likely supplied ample photosynthetic carbon and expanded the root network space, ultimately fostering the growth and reproduction of AMF. Although maize exhibits a lower mycorrhizal colonization rate than soybean, its extensive root system sustains higher HLD and SD. Therefore, designing efficient intercropping systems should combine highly mycorrhizal-responsive crops (e.g., soybean) with those possessing well-developed root systems (e.g., maize) to fully harness the ecological functions of AMF.

### 4.2 Effects of intercropping and P fertilizer level on the diversity and composition of AMF and bacterial communities

Soil environment and plant diversity are critical predictors of fungal diversity and abundance (Ma et al., 2023). Our results demonstrate that P application significantly enhanced AMF diversity (Shannon index) in both MM and IMS, while that of MS displayed a unimodal trend (**Figure 4a**). For richness index (Ace), IMS showed a continuous increase with higher P fertilizer inputs; whereas unimodal trends were observed in both monocropping systems (**Figure 4b**). This interpretation is supported by the existing literature. This discrepancy in responses is likely attributable to the synergistic effects of crop-specific P demand and mycorrhizal dependence. Previous studies have reported positive effects of P application on AMF, with higher soil P availability correlating with increased AMF diversity and richness (Da Silva et al., 2021). In this study, the same results were observed in IMS. Furthermore, the enhancement of mycorrhizal colonization rates and extraradical hyphal density under P supplementation, as documented by Shrestha Vaidya et al. (2008) and Gryndler et al. (2009), further supports this finding. Conversely, other studies have shown that AMF diversity and richness decrease with increasing soil P availability (De Beenhouwer et al, 2015; Ceulemans et al., 2019; Ma et al., 2021), which is consistent with the P suppression effect observed in MS in this study. Overall, these contrasting results demonstrate that the response of the AMF community to P fertilizer inputs is collectively determined by the interplay of soil P availability, host plant species, and cropping pattern.

At the same P fertilizer level, IMS exhibited higher AMF diversity and richness than MM (except for diversity at P50), but these values were lower than those in MS (except at P250) (**Figures 4a, b**). This observation is consistent with the reported consensus that legume/cereal intercropping enhances AMF diversity and richness compared to cereal monocultures (Lu et al., 2023; Zhang et al., 2024). This is likely because the higher plant diversity in the intercropping system can drive changes in AMF diversity by altering soil properties (Gottshall et al., 2017). Our results further confirm this, as the available P content in IMS was higher than that in MM (**Figures 3a, b**). Furthermore, the plant diversity hypothesis posits that increased plant diversity provides more ecological niches for microorganisms (Waldrop et al., 2006; Hooper et al., 2000), increasing the chances of microbes finding suitable hosts and creating a heterogeneous mycorrhizal environment that supports greater AMF diversity and richness. Notably, at the P50 level, the AMF diversity (Shannon index) in IMS was lower than that in MM. This result aligns with the findings of Lian et al. (2018) in a sugarcane/soybean intercropping system, which confirmed that the decrease in soil pH induced by intercropping was a key driver leading to the reduction of the fungal Shannon index in the rhizosphere of intercropped sugarcane.

Rhizosphere microbial communities, particularly AMF and bacteria, play a critical role in plant nutrient cycling (Zhou et al., 2022; Vejan et al., 2016). This study revealed that P fertilizer level and cropping systems markedly shaped the composition of the AMF community (**Figure 3**). Across all treatments, *Acaulospora* and *Glomus_f_Glomeraceae* were the dominant genera. This dominance may be attributed to the ecological adaptability of these two taxa: *Acaulospora* prefers acidic soil environments (Gai and Liu, 2003; Powell et al., 2009), while *Glomus_f_Glomeraceae* is commonly a dominant genus in IMS (Zhang et al., 2024). Soil AP is a key factor influencing the composition of AMF communities in farmland ecosystems (Lang et al., 2022; Wang et al., 2024). In both MM and IMS, the relative abundance of *Acaulospora* exhibited an “initial decline followed by an increase” with increasing P fertilizer inputs, reaching its minimum at P150. Conversely, the abundance of *Glomus_f_Glomeraceae* showed an “initial increase followed by a decline”, peaking at the P150 treatment. By comparison, with increasing P fertilizer inputs, MS triggered a continuous decline in *Acaulospora* and a steady rise in *Glomus_f_Glomeraceae* (**Figures 5a, b**). These divergent responses suggest differentiated P adaptation strategies of AMF across various cropping systems. Intercropping systems reshape AMF community structure through interspecific interactions and functional complementarity between crops (Zhang et al., 2020, 2024). Compared with MM, IMS reduced the relative abundance of *Acaulospora* (except at P50) while increasing that of *Glomus_f_Glomeraceae* (**Figures 5a, b**), a trend consistent with previous findings (Zhang et al., 2024). This study further revealed that, apart from a negative correlation with HLD in MS, the relative abundance of *Glomus_f_Glomeraceae* was positively correlated with mycorrhizal colonization rate, HLD, and SD (**Figure 5**). This can be primarily attributed to the members of the genus *Glomus*, which exhibit stronger root and rhizosphere colonization potential, as well as higher sporulation rates (Oehl et al., 2003; Powell et al., 2009). The abundance of these *Glomus* taxa directly determines the levels of these mycorrhizal symbiotic indicators (Wang et al., 2025b). Thus, the enrichment of highly efficient symbiotic AMF taxa (e.g., *Glomus_f_Glomeraceae*) in the intercropping system is the key reason why higher mycorrhizal colonization benefits were achieved compared to monoculture systems, ultimately resulting in an optimal state of mycorrhizal symbiosis under the P150 treatment.

Numerous studies demonstrate that soil P availability modulates bacterial community composition (Ran et al., 2021; Cao et al., 2024; Liu et al., 2024), yet its effects are highly variable across ecosystems due to differences in vegetation and edaphic conditions (Li et al., 2019; Dai et al., 2020). For instance, short-term (3-year) P fertilization had negligible effects on soil bacterial diversity and composition in tropical rainforests (Li et al., 2019). In another system, the Shannon index and relative abundance of bacterial communities in wheat rhizosphere soil decreased significantly with increasing P fertilizer application, although this effect was absent in bulk soil (Liu et al., 2024). This context-dependent variability is partially consistent with our findings. This study revealed that P fertilizer input and IMS significantly influenced bacterial community composition (**Figure 5b**). With increasing P fertilizer inputs, the bacterial diversity in MM decreased significantly, but no significant effects were observed in IMS and MS. Concurrently, the bacterial richness in both MM and MS showed a consistent decline, while IMS exhibited an initial increase followed by a decrease (**Figures 4c, d**). This discrepancy may originate from the distinct root exudate profiles of different crop types and their interaction patterns with AMF, which collectively shape a differentiated rhizosphere microenvironment, thereby mediating the specific responses of the bacterial community to P fertilizer levels. Our results demonstrate that IMS increased the rhizosphere soil AP content (**Figures 3a, c**). This finding is in line with the mechanism proposed by Ma et al. (2024), which suggests that intercropping enhances AP content by reshaping the soil bacterial community. At the same P fertilizer levels, the bacterial diversity in the rhizosphere soil of IMS (except at P50) was significantly higher than that in MM, and its richness was also higher than that in both MM and MS, although not significantly (**Figures 4c, d**). This finding is consistent with previous reports that legume/cereal intercropping enhances microbial diversity (Sun et al., 2022; Duchene et al., 2017). Moreover, the absence of a significant effect of IMS on bacterial richness suggests a relative stability of bacterial alpha diversity (Yu et al., 2021).

### 4.3 Intercropping alters interactions between AMF and bacterial taxa, facilitating P solubilization

The interaction between AMF and bacteria serves as a common and critical strategy for activating insoluble soil P (Duan et al., 2024; Jin et al., 2024; Wang et al., 2023a). This study demonstrates that IMS increased the frequency of cooperative linkages between AMF and bacteria across various P levels (**Supplementary Table S3**), suggesting that intercropping strengthens their mutually beneficial relationships and consequently facilitates P activation in rhizosphere red soil. The underlying mechanism may be driven by increased HLD and SD under IMS. On one hand, the mycelium recruits bacteria carrying the *gcd* (encoding glucose dehydrogenase) and *phoD* (encoding alkaline phosphatase [ALP]) genes, a process that synergistically drives the activation of organic P in the hyphosphere soil (Wang et al., 2023b).On the other hand, as reported by Agnolucci et al. (2015), AMF spore surfaces readily enrich PSB taxa such as Arthrobacter and Streptomyces. We therefore speculate that the increased SD in IMS may provide new colonization niches for these beneficial PSB taxa, consequently enhancing the system’PAC. Molecular evidence supports this mechanism: IMS has been demonstrated to upregulate key P transformation genes in the rhizosphere, including *phoD* (encoding ALP) for organic P mineralization (Wang et al., 2025), and *ppa/ppx* (encoding inorganic pyrophosphatase and exopolyphosphatase, respectively) for inorganic P dissolution (Tanuwidjaja et al., 2021). It is noteworthy that both the *phoD* and *ppa* genes are widely distributed across bacterial taxa such as Proteobacteria, Firmicutes, and Actinobacteria, whereas the *ppx* gene has been found exclusively in Proteobacteria (Siles et al., 2022). This distribution pattern suggests that the upregulation of these genes under intercropping may not only involve responses from multiple bacterial groups but also indicate potential functional specialization within the microbial community. However, as P fertilizer inputs increased, the proportion of positive correlations between AMF and bacteria exhibited a declining trend in both IMS and MS. This may be attributed to the fact that IMS elevates available P levels in the rhizosphere, which in turn reduces plant carbon allocation to AMF (Nagy et al., 2009). The resulting carbon limitation suppresses hyphal growth and sporulation, ultimately diminishing opportunities for interaction with bacteria. On the other hand, high P conditions weaken the relationship between the relative abundance of bacterial phyla and the abundance of the P mineralization functional gene (*phoD*) (Zhou et al., 2022).

The composition and function of the rhizosphere microbial community are regulated by root and mycorrhizal exudates (Zhalnina et al., 2018; Sasse et al., 2018; Zhang et al., 2016a). Under low-P stress conditions, the role of AMF becomes especially critical (Zhou et al., 2022). Our results demonstrate that IMS significantly alters the structure of the rhizosphere microbial community, as primarily reflected by an increase in the relative abundance of eight key bacterial taxa (**Figure 8**). Most of these enriched bacterial taxa are known to possess phosphate-solubilizing functions. Among these, *Sphingomonas* is the most abundant bacterial genus in the rhizosphere soil (**Figure 8a**, **Supplementary Figure S2**). It is also a dominant taxon in the hyphosphere (Zhou et al., 2020) and demonstrates high efficacy in degrading organic P compounds to release soluble phosphate (Midekssa et al., 2016). *Unclassified_f:Micrococcaceae* was also consistently enriched in the rhizosphere soil (**Figure 8b**). This bacterial genus has been demonstrated to dissolve mineral-bound P by secreting organic acids (Bolo et al., 2021; Mander et al., 2012). AMF can significantly enrich the genera *Streptomyces* and *Gemmatimonas* under P stress conditions (Zhou et al., 2020; Wang et al., 2023b). Their secreted ALP can mineralize organic P (Zhang et al., 2018b; Wei et al., 2019). Furthermore, *Bradyrhizobium*, in addition to its role in soil nitrogen cycling, can also dissolve inorganic P by producing phosphatases and organic acids (Deng et al., 2024; Vyas and Gulati, 2009). Emerging evidence has demonstrated that *Massilia* serves as a key member within the hyphosphere core microbiome, exhibiting remarkable P solubilization efficiency via phosphatase exudation (Wang et al., 2025a). Currently, there is no documented evidence in the literature indicating direct phosphate-solubilizing activity in either *Nocardioides* or *Lysobacter*. Based on the enrichment of these PSB taxa in the rhizosphere, we speculate that the core microbiome enhances P availability synergistically, primarily through direct pathways (e.g., secretion of phosphatases and organic acids) and indirect pathways (e.g., interactions with noncore microbial members). Therefore, we recommend adopting an “optimized P fertilization combined with legume/cereal intercropping” cultivation model in red soil regions. This approach can enhance microbial functions to sustainably improve P availability while reducing P fertilizer inputs, ultimately achieving synergistic outcomes in crop productivity and agricultural ecosystem health.

A primary limitation of this study is the inability to accurately quantify the respective contributions of root hairs and AMF (including hyphae and spores) to the recruitment of PSB in the rhizosphere. Future studies should employ single-cell fluorescence tracing combined with triple-isotope labeling (using ¹³C for root hairs, _15_N for AMF hyphae, and _18_O for AMF spores), integrated with nanoscale secondary ion mass spectrometry (NanoSIMS), high-throughput microscopic imaging, as well as metabolomics coupled with isotope labeling. Such integrated approaches would help elucidate the spatial partitioning and metabolic mechanisms underlying root hair–AMF interactions during rhizosphere microbiome assembly.

## 5 Conclusions

In summary, our study demonstrates that IMS enhances the diversity and richness of AMF in the rhizosphere soil and drives a significant increase in the relative abundance of the key functional taxon *Glomus_f_Glomeraceae*. This practice also promotes higher mycorrhizal colonization rates, HLD, and SD, while concurrently enriching eight pivotal bacterial taxa. Notably, at the P150 level, the relative abundance of *Glomus_f_Glomeraceae* reached its peak, corresponding to an optimal AMF–crop symbiotic relationship. Cross-domain co-occurrence network analysis revealed a greater prevalence of cooperative relationships between AMF and bacteria in the intercropping system compared to the monoculture, uncovering the mechanism through which intercropping promotes P activation in red soil via strengthened AMF–PSB synergistic interactions. This study establishes 150 mg P_2_O_5_ kg^−1^ dry soil as the P fertilization threshold in IMS systems. This finding not only provides a practical agricultural management strategy for red soil regions in Southwest China but also advances the theoretical framework for regulating farmland P cycling from the perspective of microbial interactions.

## Data Availability

The datasets presented in this study can be found in online repositories. The names of the repository/repositories and accession number(s) can be found below: https://www.ncbi.nlm.nih.gov/, PRJNA1236893.
