# Supplementary material for: Maize/soybean intercropping facilitated phosphorus solubilization via shifted and synergistic arbuscular mycorrhizal fungal and bacterial communities in red soil
Source: Front Plant Sci. 2025 Nov 26;16:1638043. doi: 10.3389/fpls.2025.1638043 (PMC12689951; doi:10.3389/fpls.2025.1638043)
Supplement: Supplementary file 1 [file DataSheet1.pdf]

## Supplementary Table S1-3

**Table S1** The basic physiochemical properties of soil

| pH | organic matter g/kg | total P g/kg | Olsen-P mg/kg | Total N g/kg | Available N mg/kg | total K g/kg | slow-release K mg/kg | rapidly available K mg/kg |
|---|---|---|---|---|---|---|---|---|
| 5.56 | 10.15 | 0.29 | 5.50 | 0.50 | 56.15 | 15.8 | 136.07 | 79.02 |

**Table S2** Experimental treatments design

| cropping systems | P level | | | | | |
|---|---|---|---|---|---|---|
| | P0 | P50 | P100 | P150 | P200 | P250 |
| MM | P0MM | P50MM | P100MM | P150MM | P200MM | P250MM |
| IMS | P0IMS | P50IMS | P100IMS | P150IMS | P200IMS | P250IMS |
| MS | P0MS | P50MS | P100MS | P150MS | P200MS | P250MS |

**Table S3** Topological properties of the interdomain co-occurrence network between AMF and bacterial communities

| Network metrics | P50 | | | P150 | | | P250 | | |
|---|---|---|---|---|---|---|---|---|---|
| | MM | IMS | MS | MM | IMS | MS | MM | IMS | MS |
| Nodes of bacteria | 199 | 199 | 199 | 199 | 199 | 199 | 199 | 199 | 199 |
| Nodes of AMF | 42 | 45 | 42 | 45 | 43 | 47 | 40 | 45 | 41 |
| Edges | 1522 | 1606 | 1525 | 1562 | 1539 | 1667 | 1489 | 1527 | 1569 |
| Proportion of positive edges | 59.33% | 54.23% | 56.92% | 53.59% | 56.27% | 58.37% | 54.60% | 50.88% | 53.09% |
| Proportion of negative edges | 40.67% | 45.77% | 43.08% | 46.41% | 43.73% | 41.63% | 45.40% | 49.12% | 46.91% |
| Average degree | 12.631 | 13.164 | 12.656 | 12.803 | 12.719 | 13.553 | 12.46 | 12.516 | 13.075 |
| Network density | 0.053 | 0.054 | 0.053 | 0.053 | 0.053 | 0.055 | 0.052 | 0.052 | 0.055 |
| Clustering coefficient | 0.761 | 0.758 | 0.749 | 0.786 | 0.749 | 0.743 | 0.773 | 0.752 | 0.752 |
| Network diameter | 25 | 24 | 26 | 46 | 25 | 48 | 45 | 25 | 35 |
| Average path length | 12.502 | 11.718 | 12.933 | 14.712 | 12.118 | 14.569 | 15.468 | 12.377 | 10.459 |
| Modularity | 0.803 | 0.83 | 0.822 | 0.786 | 0.807 | 0.767 | 0.849 | 0.822 | 0.815 |
| Number of modules | 10 | 10 | 9 | 10 | 10 | 9 | 9 | 10 | 8 |

## Supplementary Figures S1-4

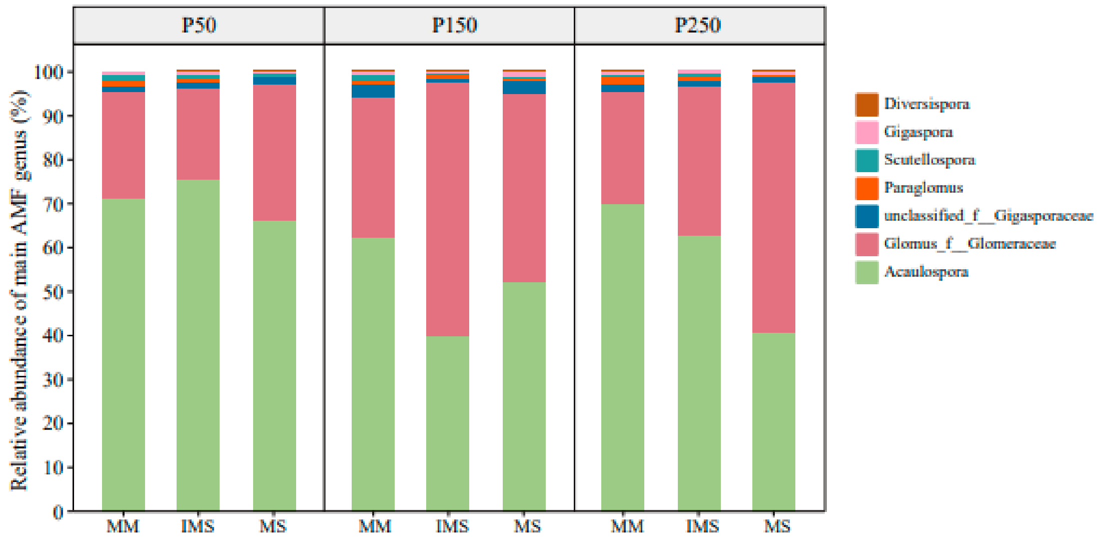


**Figure S1** Community composition of AMF genera in rhizosphere soil under different P fertilizer levels and cropping systems.

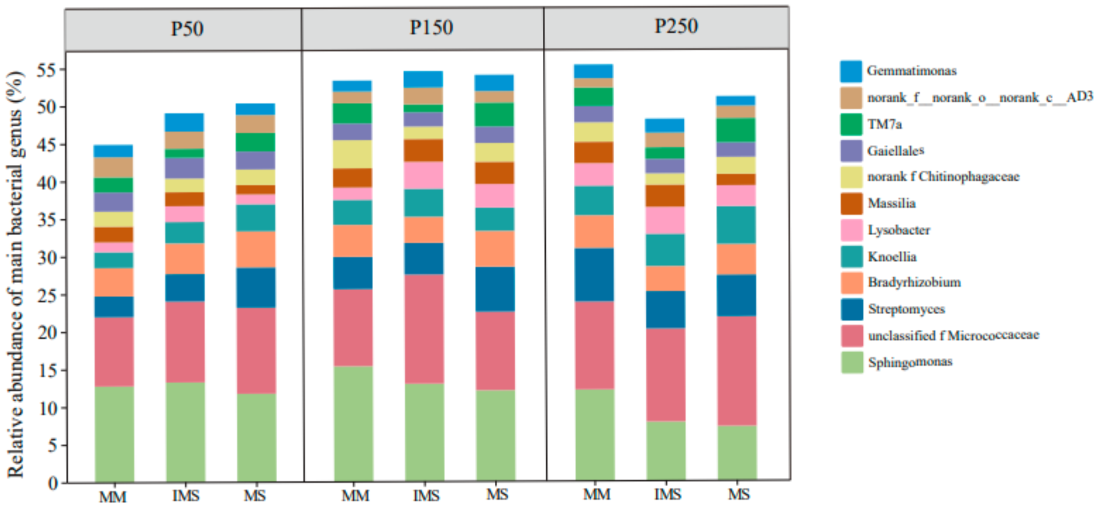


**Figure S2** Community composition of bacterial genera in rhizosphere soil under different P fertilizer levels and cropping systems.

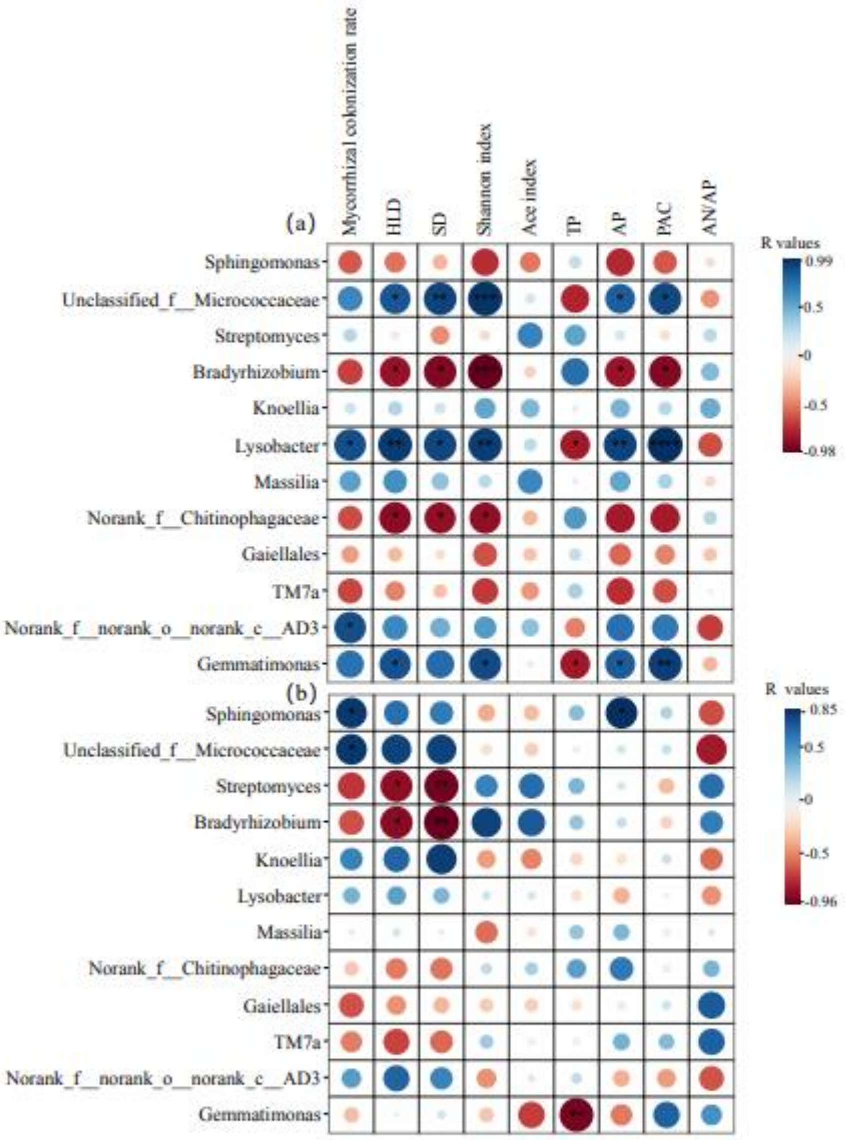


**Figure S3** Pearson correlation heatmap. Analysis between the relative abundance of the top twelve bacterial genera and AMF parameters (diversity indices, colonization rate, HLD, SD) as well as soil chemistry (TP, AP, PAC, AN/AP) in the rhizosphere soil of maize (a) and soybean (b) under P150 conditions. The color and size of the circles represent the value of the correlation coefficient (R). *$p < 0.05$; **$p < 0.01$; ***$p < 0.001$.

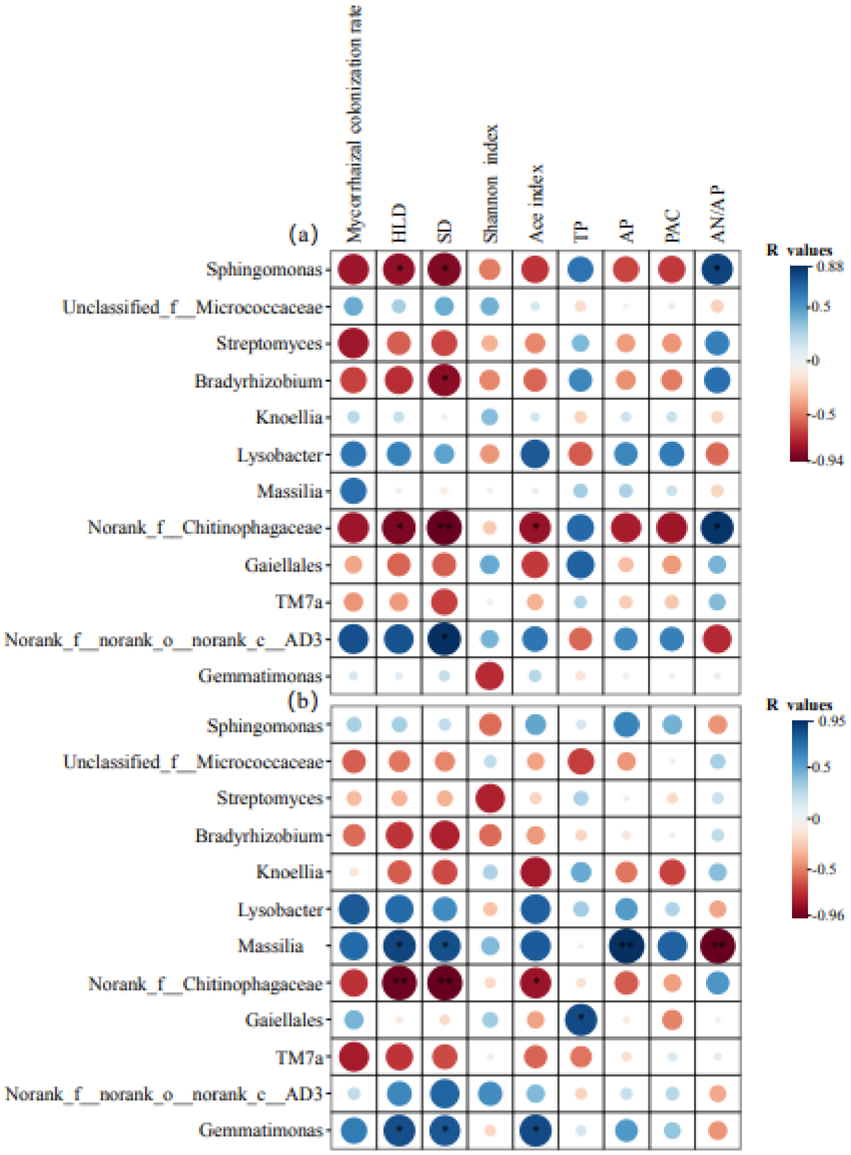


**Figure S4** Pearson correlation heatmap. Analysis between the relative abundance of the top twelve bacterial genera and AMF parameters (diversity indices, colonization rate, HLD, SD) as well as soil chemistry (TP, AP, PAC, AN/AP) in the rhizosphere soil of maize (a) and soybean (b) under P250 conditions. The color and size of the circles represent the value of the correlation coefficient (R). * $p < 0.05$; ** $p < 0.01$; *** $p < 0.001$.
